# Serum 25-Hydroxyvitamin D Levels in Type 2 Diabetes Patients in North China: Seasonality and the Association between Vitamin D Status and Glycosylated Hemoglobin Levels

**DOI:** 10.1155/2023/4151224

**Published:** 2023-05-05

**Authors:** Chang Wang, Huan Li, Lijing Huo, Qing Wang, Tian Zhang, Xiaoyu He, Jianan Hao, Yu Luo, Luping Ren

**Affiliations:** ^1^Department of Endocrinology, Hebei General Hospital, Shijiazhuang 050051, China; ^2^Graduate School of Hebei Medical University, Shijiazhuang 050017, China; ^3^Department of Clinical Laboratory, Hebei General Hospital, Shijiazhuang 050051, China

## Abstract

**Background and Aims:**

Previous studies have reported a correlation between vitamin D levels and seasonality in healthy populations. However, there are few studies on the seasonal variation in vitamin D levels and its relationship with glycosylated hemoglobin (HbA1c) in patients with type 2 diabetes mellitus (T2DM). The objective of this study was to investigate seasonal changes in serum 25-hydroxyvitamin D [25(OH)D] levels and the associations between these vitamin D concentrations and HbA1c levels in T2DM patients in Hebei, China.

**Methods:**

A cross-sectional study of 1,074 individuals with T2DM was conducted from May 2018 to September 2021. Levels of 25(OH)D in these patients were assessed based on both sex and season, and relevant clinical or laboratory variables that could impact vitamin D status were also considered.

**Results:**

In the T2DM patient cohort, the mean blood 25(OH)D levels were 17.05 ng/mL. A total of 698 patients (65.0%) had insufficient serum 25(OH)D levels. The vitamin D deficiency rates were significantly higher in the winter and spring compared to the autumn (*P* < 0.05), indicating that seasonal fluctuations can have a significant impact on 25(OH)D levels. The levels of vitamin D inadequacy were highest in the winter (74%), and females were more likely than males to be deficient (73.4% vs. 59.5%, *P* < 0.001). In comparison to the winter and spring, both males and females showed higher 25(OH)D levels in the summer (*P* < 0.001). HbA1c levels were 8.9% higher in those with vitamin D deficiencies than in nondeficient patients (*P* < 0.001). HbA1c and vitamin D levels were negatively correlated (*r* = −0.119, *P* < 0.001).

**Conclusion:**

Vitamin D deficiencies are particularly prevalent among T2DM patients in Hebei, China, with exceptionally high rates in the winter and spring. Female T2DM patients were at an elevated risk of vitamin D deficiency, and vitamin D levels were negatively correlated with HbA1c.

## 1. Introduction

Vitamin D deficiency is increasingly prevalent worldwide, resulting from reduced exposure to daily sunlight and ultraviolet (UV) radiation, even in tropical regions [1, 2]. High levels of vitamin D deficiency have been reported throughout China [3]. Reduced solar UV radiation penetration is one driver of vitamin D deficiency. Rapid industrialization and urbanization in Northern China have contributed to high levels of air pollution that have impaired this UV penetration [4]. A relationship between vitamin D and the development of type 2 diabetes mellitus (T2DM) has been firmly established [5, 6]. Prior work suggests that vitamin D can influence T2DM progression by impacting the pancreatic beta cells and contributing to systemic inflammation [5, 7–9].

Seasonal changes in vitamin D levels have been reported throughout the world [10–14]. Only relatively low levels of solar UV radiation reach the earth's surface in the winter, contributing to this seasonality [15]. Indeed, vitamin D levels tend to be lowest in the winter and highest in the summer in mid- and high-latitude regions such as China, Germany, Poland, and other Northern European nations [16–22]. This seasonal shift is also evident in Australia and other low-latitude areas [23], indicating that season rather than latitude is the primary driver of this variation [24]. China's Hebei Province, which encircles Beijing, is situated in a moderate climate region with noticeable seasonal variations between longitudes 113°27′ and 119°50′ East and latitudes 36°05′ and 42°40′ North. Severe vitamin D deficiency rates among residents of different age groups in Beijing have been reported to be high, particularly in the winter and spring [22]. Similar results have been observed in specific studies of the elderly and women suffering from rheumatic diseases [25, 26]. However, studies exploring seasonal shifts in vitamin D levels in T2DM patient populations are currently lacking.

Prior studies have shown that the glycosylated hemoglobin (HbA1c) levels in T2DM are correlated with vitamin D concentrations [27, 28]. In particular, Buhary et al. discovered a negative correlation between HbA1c levels and serum vitamin D concentrations in diabetic patients, with concentrations decreasing after vitamin D administration [29]. By examining the relationship between vitamin D status and HbA1c levels in 238 Chinese T2DM patients in 2020, our team identified a connection between vitamin D insufficiency and high HbA1c levels in this patient sample [30]. Additionally, multiple studies have demonstrated a correlation between glycemic control and seasonality, as in the case of studies from Japan, Korea, and Portugal in which T2DM patient HbA1c levels were reported to be higher during the colder months [31–33]. Korea is located between the latitudes of 34°N and 38°N, while Japan is between 20°N and 46°N, and China falls within different latitudes. No published studies have examined seasonal shifts in HbA1c levels among Chinese T2DM patients.

There are few studies on the relationship between seasonal fluctuations in vitamin D levels and HbA1c levels in people with T2DM. Therefore, the goal of this research was to determine seasonal changes in serum 25(OH)D levels and the relationship between these vitamin D levels and HbA1c levels in T2DM patients in Hebei, China.

## 2. Materials and Methods

### 2.1. Patient Recruitment

This study included 1,074 patients with T2DM who were evaluated at the Hebei General Hospital in Shijiazhuang, China, between May 4, 2018, and September 3, 2021, with matching based on sex and season. This study received approval from the Hebei General Hospital Ethics Committee (Number: 2022156), and all patients provided informed consent. The study was in accordance with the Declaration of Helsinki. Two investigators who established consensus criteria for patient inclusion/exclusion independently evaluated the study's feasibility.

Individuals eligible for inclusion were T2DM patients meeting the 1999 World Health Organization (WHO) diagnostic criteria. Samples of venous blood were collected from these patients to assess blood glucose levels, and individuals free of diabetic symptoms underwent repeat blood draws on a separate day. Patients were excluded from this analysis if they had been diagnosed with hyperparathyroidism, severe renal, liver, or intestinal diseases or were consuming supplemental calcium or vitamin D.

### 2.2. Methods

Primary T2DM patient demographic characteristics such as age, sex, diabetes duration, and body mass index (BMI) were obtained through electronic medical records and a questionnaire. Mean systolic and diastolic blood pressure (SBP and DBP, respectively) were assessed with a sphygmomanometer. Samples of venous blood were collected from all patients after an overnight fasting interval of at least 8 hours. Levels of lipids, calcium, phosphorus, 25-hydroxyvitamin D (25[OH]D), parathyroid hormone (PTH), glucose metabolism-related indicators (fasting blood glucose (FBG), HbA1c), and bone turnover markers (osteocalcin (OC), *β*-C-terminal cross-linked telopeptide of type I collagen (*β*-CTX), and procollagen type 1 N-terminal propeptide (P1NP)) were analyzed using these samples. The resultant data were incorporated into a spreadsheet, and correct data input was verified by two investigators. Serum 25(OH)D was quantified using a commercially available electrochemiluminescence immunoassay (ECLA) kit (COBAS e 601, Roche, Germany).

### 2.3. Statistical Analyses

SPSS 26.0 (IBM Corp., Armonk, NY, USA) and GraphPad Prism 8.0 (GraphPad Software, San Diego, CA, USA) were used to conduct all statistical analyses. Serum 25(OH)D levels of <20 ng/mL (<50 nmol/L) were used to define vitamin D deficiency [34]. As solar radiation exposure influences vitamin D status, seasons were determined based on standard dates for the northern hemisphere (Spring: March 21-June 20; Summer: June 21-September 22; Fall: September 23-December 21; Winter: December 22-March 20) [22].

Data comparisons were made for the following groups: (1) data were compared for samples collected in each of the four seasons; (2) data were compared between patients who were and were not vitamin D-deficient; (3) percentages of vitamin D deficiency in each season were compared; (4) vitamin D deficiency percentages were compared between males and females; (5) percentages of vitamin D deficiency were compared among seasons and sexes, and (6) male and female 25(OH)D levels were compared for each season. All data were examined to assess whether they fit into a normal distribution. Medians and interquartile ranges (P25, P75) were utilized to express nonnormally distributed continuous data. The proper one-way ANOVA or nonparametric tests were used to compare these data. Chi-square tests were used for the analysis of categorical data presented as numbers (percentages). A linear correlation analysis was used to compare the relationships between serum 25(OH)D and HbA1c levels. Linear regression analysis was performed based on three different models (model 1: crude model; model 2: partial adjustment for general variables; model 3: adjustment for all relevant variables) to determine whether 25(OH)D levels independently affected HbA1c levels. The level of significance was established at a two-tailed *p* < 0.05.

## 3. Results

### 3.1. Participant Characteristics

In total, 1,074 T2DM patients were recruited between May 4, 2018, and September 3, 2021. Patient demographic and biochemical features are shown in Table 1. Of these patients, 39.6% were female, and the median population age was 57 years (IQR 49–66). Overall, 36.0% of these individuals had been diagnosed with T2DM for over 10 years. The median HbA1c in this cohort was 8.7 (IQR 7.4–10.4) %, and the median BMI was 26.20 (IQR 23.78-28.68) kg/m^2^.

### 3.2. Patient Vitamin D Status Characteristics

**Figure 1:**
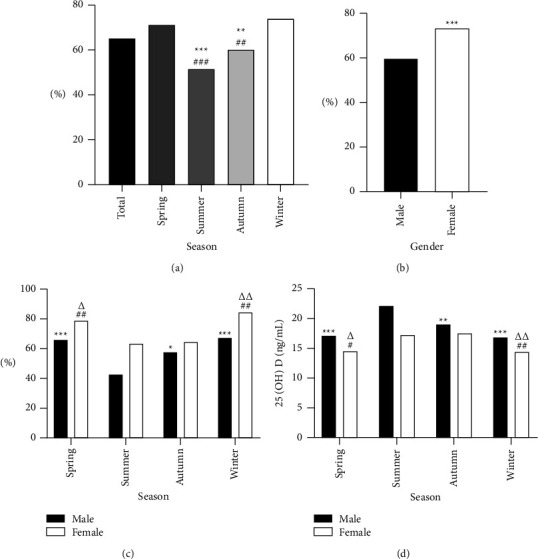
Percentage of vitamin D deficiency and vitamin D levels according to season and sex in T2DM. (a) The percentage of vitamin D deficiency in total population and different seasons, compared with spring, ^*∗∗∗*^*p* < 0.001, ^*∗∗*^*p* < 0.01;compared with winter, ^###^*p* < 0.001, ^##^*p* < 0.01; (b) the percentage of vitamin D deficiency according to sex, ^*∗∗∗*^*p* < 0.001; (c) the percentage of vitamin D deficiency in different seasons and sexes. In males, compared with summer, ^*∗∗∗*^*p* < 0.001, ^*∗*^*p* < 0.05. In females, compared with summer, ^##^*p* < 0.01, ^#^*p* < 0.05;compared with autumn, ^△△^<0.01, ^△^<0.05; (d) male and female subjects in this study were divided into four groups according to serum 25(OH)D level testing season. In males, compared with summer, ^*∗∗∗*^*p* < 0.001, ^*∗∗*^*p* < 0.01. In females, compared with summer, ^##^*p* < 0.01, ^#^*p* < 0.05; compared with autumn, ^△△^<0.01, ^△^<0.05.

In the overall study cohort, the mean serum 25(OH)D levels were 17.05 (IQR 13.58-22.39) ng/mL, with a high incidence of vitamin D deficiency in the cohort, affecting 65.0% of these individuals. The frequency of vitamin D deficiency was highest in the winter (74.0%, *p* < 0.01) and lowest in the summer (51.6%, *p* < 0.01), with respective prevalence rates of 71.0% and 60.3% in spring and fall (Figure 1(a)). Incidence rates were significantly higher among women (73.4%) as compared to men (59.5%, *p* < 0.01) (Figure 1(b)). However, similar seasonal trends in vitamin D deficiency rates were observed in both males and females. Specifically, the vitamin D deficiency rates among males in the summer were lower than in the three other seasons, while in women, these rates were lower in the summer and autumn relative to the spring and winter. Serum 25(OH)D levels thus tended to be higher in the autumn and summer relative to the winter and spring, and the same seasonal trend remained significant in both males and females (*p* < 0.05). The summer was the only season in which men exhibited a mean 25(OH)D level >20 ng/mL, with lower levels in all three other seasons. In women, 25(OH)D levels in the summer and autumn were higher than those in the spring and winter (Figures 1(c) and 1(d)). As such, it is important that 25(OH)D levels be monitored irrespective of the season, such monitoring is particularly important for women in the winter and spring.

### 3.3. Seasonal Variations in Serum 25(OH)D Levels and Other Analyzed Clinical Parameters

The mean 25(OH)D levels in this cohort of T2DM patients were 16.31 (IQR 12.97–21.14) ng/mL in the spring, 19.60 (IQR 15.44–25.52) ng/mL in the summer, 18.28 (IQR 14.00–23.06) ng/mL in the fall, and 15.84 (IQR 12.31-20.36) in the winter. Nonparametric tests revealed significant differences in 25(OH)D levels among these seasons (*p*=0.000). Significant differences in other clinical variables were also observed when comparing the four seasons, including patient SBP, DBP, serum parathyroid hormone (PTH), apolipoprotein A1 (ApoA1), osteocalcin (OC), triiodothyronine (TT3), tetraiodothyronine (TT4), and free thyroxine (FT3) levels. Post-hoc multiple comparisons testing revealed that patient 25(OH)D levels in the summer and autumn were significantly higher than those in the spring and winter (*p* < 0.05), even though all levels were below the established threshold for 25(OH)D deficiency (<20 ng/mL) [34]. Levels of OC in the summer were higher than those in the autumn (*p*=0. 026), while serum PTH concentrations in the spring were elevated compared to the other three seasons (*p* < 0.05). Moreover, mean SBP and DBP values in the winter were elevated compared to the summer (*p* < 0.05), and levels of TT3 and TT4 were lower in the winter on average compared to the three other seasons (*p* < 0.05), while the highest FT3 levels were measured in the spring and summer (*p* < 0.05) (Table 1).

### 3.4. Clinical Characteristics of Vitamin D-Deficient and Nondeficient Patients

**Figure 2:**
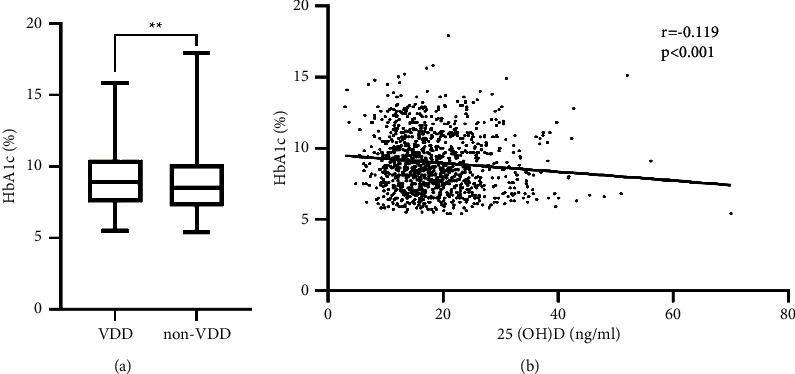
HbA1c levels according to vitamin D status and linear correlation of HbA1c and 25(OH)D in type 2 diabetic patients. (a) The patients were divided into VDD and non-VDD groups according to vitamin D levels. HbA1c levels were compared between the two groups, ^*∗∗*^: *p* < 0.01; (b) linear correlation between HbA1c and 25(OH)D in type 2 diabetic patients.

Patients were next grouped into individuals who were vitamin D deficient (VDD) (25(OH)D <20 ng/mL; *n* = 698) and vitamin D nondeficient (VDND) (25(OH)D >20 ng/mL; *n* = 376). Individuals in the VDD group exhibited significantly elevated BMI (*p*=0.037), phosphorus (*p*=0.039), triglyceride (TG) (*p*=0.018), collagen type 1 N-terminal propeptide (P1NP) (*p*=0.017), PTH (*p*=0.000), and HbA1c (*p*=0.006, Figure 2(a)) levels as compared to VDND patients, whereas VDND individuals exhibited significantly lower albumin and FT3 levels (both *p* < 0.05). No differences were observed between these groups with respect to age, T2DM duration, total cholesterol (TC), low-density lipoprotein cholesterol (LDL-C), high-density lipoprotein cholesterol (HDL-C), ApoA1, apolipoprotein b (Apob), FBG, OC, beta-crosslinked C-terminal telopeptide of collagen I (*β*-CTX), phosphorus, TT3, TT4, free tetraiodothyronine (FT4), or thyroid stimulating hormone (TSH) levels (Table 2).

### 3.5. Analysis of the Correlations between Serum 25(OH)D Levels and Other Clinical Parameters

Serum 25(OH)D levels were significantly negatively correlated with BMI (*r* = −0.068, *p*=0.026), HbA1c (*r* = −0.119, *p* < 0.001), SBP (*r* = −0.060, *p*=0.049), and TG (*r* = −0.069, *p*=0.024) levels, while they were positively correlated with serum calcium (*r* = 0.108, *p* < 0.001) and FT3 levels (*r* = 0.143, *p* < 0.001) (Figure 2(b), Table 3).

### 3.6. Linear Regression Analysis of Serum 25(OH)D and HbA1c

Serum 25(OH)D levels were negatively correlated with HbA1c before adjustment for potential confounders. The same results were obtained when some or all potential confounders were adjusted (*P*  <  0.01), which indicated that 25(OH)D was independently associated with HbA1c (Table 4).

## 4. Discussion

To the best of our knowledge, this is the first study to have specifically focused on seasonal variations in serum vitamin D in patients with T2DM. The results indicated that 65% of the analyzed subjects in Hebei, China (northern latitude: 36°05′–42°40′), were affected by vitamin D deficiency, with these rates being over 50% during all four seasons. Additionally, serum vitamin D concentrations were increased during the summer and autumn relative to the spring and winter among patients with T2DM.

Both latitude and season can impact UV radiation effectivity, thus governing the synthesis of vitamin D. In a previous study performed by Ning et al. examining the vitamin D status of residents in Beijing at a similar latitude to that analyzed in our study, an 87.1% vitamin D deficiency rate was observed in their study population, with higher rates during the winter and spring relative to the summer and autumn [22]. Other studies have also assessed the seasonality of vitamin D deficiency. For example, Karacan et al. assessed the serum 25(OH)D levels of healthy individuals in Istanbul (41° north latitude), revealing yearly fluctuations in these levels with a peak in late summer and declining levels in the spring [35]. A separate study of the British Biobank dataset revealed that vitamin D deficiency rates among persons of color in northern England were higher in the winter and spring, highlighting the close associations between vitamin D levels, seasonality, and geographic distributions [36]. None of these studies have specifically focused on seasonal variations in T2DM patient vitamin D levels. Here, we found vitamin D deficiency rates to be very high among T2DM patients during all four seasons, with seasonal variations comparable to those in the general population.

Vitamin D levels also reportedly differ between males and females. Here, the serum 25(OH)D levels in women were lower than those in men during all seasons other than autumn. Previously, Mariam et al. determined that 77% of women with T2DM were affected by vitamin D deficiency and that 78% exhibited poor glycemic control [37]. A hospital-based analysis performed by Sheth et al. in Gujarat in Western India revealed that 91.4% of women with diabetes were vitamin D deficient [38]. In the present study, 73.4% of female T2DM patients were vitamin D deficient.

Many different variables can contribute to vitamin D deficiency rates. For one, while there is a growing body of evidence indicating that vitamin D deficiency can pose a major risk to human health, awareness of the effects of vitamin D deficiency is still relatively limited due to relatively low levels of education on this topic. Second, endogenous vitamin D production can be limited by many factors including the length of sunlight exposure, environmental pollution, regional latitude, and the use of sunscreen, as shown by worm conducted by Goswami et al. demonstrating that outdoor workers in West Bengal, India, with T2DM had very low rates of vitamin D deficiency [39]. Third, foods rich in vitamin D such as fish, seafood, and milk are not staples of Chinese diets, and foods fortified with vitamin D are not as readily available in China as they are in the West [40]. The present data also highly sex-specific differences in vitamin D levels were lower among women than men, potentially due to higher levels of concern regarding skin darkening among women and/or the thicker clothing that women often wear in the winter in China. Women in the Hebei area of China often use sunscreen and other forms of UV protection in the summer, which may contribute to a more pronounced lack of sun exposure as compared to that in men.

Here, T2DM patient 25(OH)D and HbA1c levels were found to be negatively associated. We also found that the 25(OH)D level in T2DM patients was an independent risk factor for high HbA1c levels, with or without adjustment for all confounders. This is consistent with previous findings. In studies of patients with gestational diabetes, 25(OH)D levels during oral glucose tolerance testing (OGTT) were found to be an independent predictor of HbA1c levels [41]. At the same time, vitamin D was found to be an independent influencing factor of glycosylated hemoglobin levels not only in T2DM but also in nondiabetic patients [42]. The control of blood glucose by vitamin D depends on the participation of islet cells. In prior reports, vitamin D deficiency has been shown to interfere with insulin synthesis and secretion [5, 43–45]. The pancreatic beta cells express active vitamin D receptors [5], which allows for the vitamin D-mediated modulation of insulin responses to elevated levels of blood glucose. Vitamin D is also capable of inhibiting inflammation and thereby promoting insulin-mediated reactions [46]. Given its potential to influence metabolic disease status, many researchers have conducted interventional vitamin D supplementation studies. In one case, significant decreases in both HbA1c values and fasting blood glucose relative to baseline were reported in T2DM patients that consumed 4500 IU of vitamin D per day for a two-month period [47]. The current data also indicate vitamin D's possible function as a glycemic control regulator.

We found no evidence of seasonal fluctuations in HbA1c, despite a negative connection between HbA1c and vitamin D levels. However, earlier studies have shown a significant seasonal change in the glycemic control of T2DM patients [48]. These inconsistent findings may result from variations in the latitude of the study site, ethnic differences, or other underlying baseline differences in these populations.

Here, the OC levels were found to vary seasonally, being highest in the summer, while blood calcium, *β*-CTX, and P1NP did not differ significantly between the different seasons. No significant differences in blood calcium or *β*-CTX levels were observed in the VDD and VDND groups, and further investigation will be needed to probe the underlying mechanisms. PTH levels in this analysis were higher in the winter and spring relative to the summer and autumn, with higher PTH levels in the VDD patient cohort relative to VDND patients. Serum 25(OH)D levels were negatively associated with P1NP and PTH levels and were positively related to blood calcium levels. This suggests the possibly of bone metabolism disturbances in the vitamin D-deficient group.

TG levels in the T2DM patients analyzed in this study were higher in VDD patients relative to VDND individuals, and TG and vitamin D levels were negatively correlated (*r* = −0.069, *p*=0.024). Mechanistically, this may suggest that TG levels correlate with vitamin D status as a consequence of the ability of vitamin D to impact insulin sensitivity. Vitamin D deficiency can alter the function of pancreatic beta cells in a manner conducive to insulin resistance, disrupting lipoprotein metabolism and thereby increasing the levels of TG in systemic circulation [49]. Vitamin D can also have a direct impact on lipid metabolism and the hepatic synthesis of bile acid [50]. The link between dyslipidemia and vitamin D deficiency may thus be multifactorial. A prior Korean study also found TG and vitamin D levels to be negatively correlated, while also observing a negative relationship between vitamin D and both LDL-C and TC [51]. These latter correlations were not detected in the present study, with the inconsistency potentially being attributable to differences in the dietary composition of baseline characteristics of these populations.

In this patient population, serum 25(OH)D levels were negatively associated with SBP, and both SBP and DBP levels were higher in the winter than in summer. According to these results, Kota et al. had stated previously increased SBP, DBP, and mean arterial pressure levels in vitamin D-deficient people, demonstrating a connection between this condition and the antagonism of the renin-angiotensin-aldosterone system (RAAS) [52]. Correlative relationships between vitamin D deficiency, hypertension, and cardiovascular disease are attributable to the presence of vitamin D receptors and the enzyme responsible for converting vitamin D into its active form in the endothelial, vascular smooth muscle, and cardiac tissue. We posit that the observed changes in T2DM patient blood pressure may be at least partially the result of seasonal vitamin D variations.

Serum vitamin D levels in this cohort were also positively correlated with FT3 levels. Notably, vitamin D has been found to correlate with higher type 2 deiodinase (DIO2) levels in a range of tissue types in diabetic model rats including the heart, brain, liver, kidneys, muscle, and femur, thereby promoting peripheral thyroxine (T4) conversion to triiodothyronine (T3) [53]. Moreover, the impact of vitamin D on the thyroid hormone spectrum is reportedly associated with the immunomodulatory, anti-inflammatory, and antioxidant effects of this vitamin [54].

There are some limitations to this analysis. For one, the study population only included Chinese T2DM patients from within a limited latitude range in Hebei Province, China, and the findings may thus not be generalizable to other populations or regions. In addition, only T2DM patients were analyzed without any corresponding healthy control group. Additionally, lifestyle factors were not taken into consideration for these analyses. Lastly, this analysis is subject to the inherent limitations of a cross-sectional study. Additional carefully constructed analyses will be necessary to examine the benefits of prophylactic supplemental vitamin D intake during different seasons to better clarify the clinical efficacy associated with such interventions among patients with T2DM.

## 5. Conclusions

In summary, the present data indicate that vitamin D deficiency rates are high among T2DM patients in Hebei Province in China, particularly during the spring and winter. Female T2DM patients may be particularly susceptible to vitamin D deficiency, and the levels of this vitamin and HbA1c levels were found to be negatively correlated with one another in T2DM patients.

## Figures and Tables

**Table 1:** Clinical characteristics of enrolled subjects.

Variables	Baseline *N* = 1074	Spring *n* = 386 (35.9%)	Summer *n* = 246 (22.9%)	Autumn *n* = 219 (20.4%)	Winter *n* = 223 (20.8%)	*p*
Age (years)	57 (49–66)	58 (48–67)	57 (48–67)	58 (52–66)	57 (49–66)	0.844
Sex						
Male (*n*, %)	649 (60.4%)	241 (62.4%)	139 (56.5%)	131 (59.8%)	138 (61.9%)	
Female (*n*, %)	425 (39.6%)	145 (37.6%)	107 (43.5%)	88 (40.2%)	85 (38.1%)	
Duration of DM (years)	9 (3–15)	8 (3–15)	10 (3–15)	9 (3–15)	8 (3–15)	0.647
BMI (kg/m^2^)	26.20 (23.78–28.68)	26.21 (23.80–28.41)	25.60 (23.44–28.65)	26.87 (24.22–29.21)	26.12 (24.00–28.68)	0.056
SBP (mmHg)	133 (122–147)	132 (121–145)	133 (120–143)^△^	133 (123–150)	136 (124–148)	**0.007**
DBP (mmHg)	81 (74–90)	81 (74–89)	79 (72–89)^△^	82 (75–90)	83 (75–91)	**0.028**
Albumin (g/L)	41.9 (39.3–44.3)	41.8 (39.4–44.2)	42.6 (39.6–44.5)	41.4 (39.4–44.2)	41.8 (38.8–44.5)	0.390
TC (mmol/L)	4.75 (3.94–5.62)	4.68 (3.88–5.63)	4.73 (3.85–5.57)	4.74 (3.93–5.58)	4.87 (4.22–5.51)	0.502
TG (mmol/L)	1.45 (1.01–2.24)	1.45 (0.99–2.15)	1.45 (1.01–2.39)	1.52 (1.09–2.32)	1.43 (1.00–2.06)	0.415
LDL-C (mmol/L)	3.09 (2.46–3.69)	3.02 (2.40–3.69)	3.10 (2.42–3.71)	3.11 (2.48–3.76)	3.17 (2.57–3.56)	0.744
HDL-C (mmol/L)	1.08 (0.92–1.26)	1.08 (0.92–1.28)	1.10 (0.93–1.26)	1.06 (0.91–1.24)	1.07 (0.92–1.26)	0.726
ApoA1 (g/L)	1.20 (1.04–1.36)	1.19 (1.01–1.34)	1.19 (1.04–1.33)	1.23 (1.08–1.39)	1.23 (1.09–1.38)	**0.011**
Apob (g/L)	0.82 (0.67–0.99)	0.81 (0.67–0.98)	0.83 (0.65–1.02)	0.81 (0.68–1.00)	0.82 (0.69–0.96)	0.984
FBG (mmol/L)	8.32 (6.55–10.89)	8.53 (6.75–11.23)	8.42 (6.41–10.83)	7.84 (6.45–10.42)	8.29 (6.59–10.81)	0.177
HbA1c%	8.7 (7.4–10.4)	8.8 (7.5–10.7)	8.8 (7.4–10.7)	8.6 (7.5–10.1)	8.6 (7.3–10.2)	0.481
25(OH)D (ng/mL)	17.05 (13.58–22.39)	16.31 (12.97–21.14)	19.60 (15.44-25.52)^△△^^*∗∗*^	18.28 (14.00–23.06)^△△^^*∗*^	15.84 (12.31–20.36)	**0.000**
OC (ng/mL)	12.74 (9.99–15.97)	12.80 (9.81–16.04)	13.16 (10.26–17.33)	11.70 (9.71–14.91)^#^	12.99 (10.27–16.49)	**0.026**
*β*-CTX (ng/mL)	0.39 (0.26–0.51)	0.39 (0.25–0.48)	0.40 (0.27–0.50)	0.42 (0.28–0.56)	0.38 (0.23–0.52)	0.107
PINP (ng/mL)	38.93 (29.75–49.48)	37.63 (28.74–46.73)	39.14 (29.47–50.64)	37.65 (30.17–49.58)	41.43 (30.99–50.47)	0.076
PTH (pg/mL)	38.32 (29.23–49.09)	41.79 (32.96–51.12)	36.52 (27.64-47.47)^*∗∗*^	34.32 (26.90–45.71)^*∗∗*^	38.16 (28.41–48.53)^*∗∗*^	**0.000**
Ca (mmol/L)	2.30 (2.22–2.37)	2.30 (2.23–2.37)	2.30 (2.22–2.38)	2.29 (2.22–2.36)	2.29 (2.21–2.36)	0.557
P (mmol/L)	1.22 (1.10–1.35)	1.19 (1.07–1.30)	1.25 (1.13–1.37)^*∗∗*^^△^	1.24 (1.13–1.37)^*∗∗*^	1.20 (1.08–1.34)	**0.000**
TT3 (nmom/L)	1.55 (1.35–1.74)	1.55 (1.35–1.76)^△^	1.59 (1.41–1.74)^△△^	1.55 (1.36–1.77)^△^	1.49 (1.27–1.67)	**0.002**
TT4 (nmom/L)	94.65 (82.97–106.62)	95.41 (82.96–107.90)^△△^	97.35 (89.16–109.15)^△△^	93.91 (81.88–106.80)^△#^	89.08 (78.21–99.89)	**0.000**
FT3 (pmom/L)	4.50 (4.02–4.88)	4.56 (4.01–4.94)^△△^	4.55 (4.19–4.94)^△△^	4.43 (4.04–4.77)	4.36 (3.88–4.78)	**0.001**
FT4 (pmol/L)	16.60 (14.87–18.13)	16.62 (14.95–18.14)	16.64 (15.13–18.11)	16.51 (14.77–18.21)	16.31 (14.42–17.92)	0.327
TSH (*μ*IU/mL)	1.90 (1.29–2.72)	1.87 (1.27–2.68)	1.92 (1.28–2.63)	1.90 (1.32–2.67)	1.95 (1.33–2.94)	0.801

*Notes*. Compared with spring, ^*∗∗*^*p* < 0.01, ^*∗*^*p* < 0.05; Compared with summer, ^##^*p* < 0.01, ^#^*p* < 0.05; Compared with winter, ^△△^*p* < 0.01, ^△^*p* < 0.05. BMI, body mass index; SBP, systolic blood pressure; DBP, diastolic blood pressure; TC, total cholesterol; TG, triglyceride; LDL-C, low-density lipoprotein cholesterol; HDL-C, high-density lipoprotein cholesterol; ApoA1, apolipoproteinA1; Apob, apolipoprotein b; FBG: fasting blood glucose; OC, osteocalcin; *β*-CTX, *β*-C-terminal cross-linked telopeptide of type I collagen; P1NP, procollagen type 1 N-terminal propeptide; PTH, parathyroid hormone; Ca, calcium; P, phosphorus; TT3, triiodothyronine; TT4, tetraiodothyronine; FT3, free thyroxine; FT4, free tetraiodothyronine; TSH, thyroid-stimulating hormone. Bold values indicate statistically significant results.

**Table 2:** Characteristics of patients with and without vitamin D deficiency.

Variables	Vitamin D deficient (*n* = 698)	Non-vitamin D deficient (*n* = 376)	*p*-value
Age (years)	57 (48–66)	59 (52–66)	0.050
Sex			
Male (*n*)	386	263	
Female (*n*)	312	113	
Duration of DM (years)	8 (3–15)	10 (3–15)	0.264
BMI (kg/m^2^)	26.46 (23.89–28.88)	25.78 (23.72–28.15)	**0.037**
SBP (mmHg)	134 (122–148)	132 (122–146)	0.159
DBP (mmHg)	81 (74–90)	81 (74–89)	0.723
Albumin (g/L)	41.57 (38.8–43.8)	42.8 (40.3–45.0)	**0.000**
TC (mmol/L)	4.77 (3.95–5.58)	4.74 (3.91–5.53)	0.754
TG (mmol/L)	1.52 (1.03–2.30)	1.39 (0.99–2.07)	**0.018**
LDL-C (mmol/L)	3.09 (2.47–3.66)	3.08 (2.43–3.73)	0.695
HDL-C (mmol/L)	1.06 (0.91–1.26)	1.10 (0.94–1.26)	0.091
ApoA1 (g/L)	1.19 (1.04–1.35)	1.22 (1.06–1.37)	0.099
Apob (g/L)	0.81 (0.67–0.99)	0.82 (0.66–0.99)	0.918
FBG (mmol/L)	8.5 (6.6–11.1)	8.0 (6.5–10.4)	0.150
OC (ng/mL)	12.85 (10.14–16.39)	12.58 (9.74–15.82)	0.088
*β*-CTx (ng/mL)	0.40 (0.26–0.52)	0.39 (0.25–0.50)	0.361
PINP (ng/mL)	40.10 (30.17–49.82)	37.09 (28.54–48.60)	**0.017**
PTH (pg/mL)	40.52 (30.80–50.14)	34.77 (26.76–45.17)	**0.000**
Ca (mmol/L)	2.30 (2.22–2.36)	2.30 (2.22–2.39)	0.130
P (mmol/L)	1.23 (1.10–1.35)	1.20 (1.08–1.32)	**0.039**
TT3 (nmom/L)	1.54 (1.34–1.73)	1.57 (1.37–1.77)	0.067
TT4(nmom/L)	93.84 (82.02–106.40)	95.99 (83.94–107.05)	0.073
FT3 (pmom/L)	4.44 (3.99–4.85)	4.57 (4.08–4.96)	**0.025**
FT4 (pmol/L)	16.59 (14.77–18.04)	16.62 (14.94–18.44)	0.366
TSH (*μ*IU/mL)	1.90 (1.26–2.74)	1.90 (1.33–2.65)	0.908

BMI, body mass index; SBP, systolic blood pressure; DBP, diastolic blood pressure; TC, total cholesterol; TG, triglyceride; LDL-C, low-density lipoprotein cholesterol; HDL-C, high-density lipoprotein cholesterol; ApoA1, apolipoproteinA1; Apob, apolipoprotein b; FBG: fasting blood glucose; OC, osteocalcin; *β*-CTX, *β*-C-terminal cross-linked telopeptide of type I collagen; P1NP, procollagen type 1 N-terminal propeptide; PTH, parathyroid hormone; Ca, calcium; P, phosphorus; TT3, triiodothyronine; TT4, tetraiodothyronine; FT3, free thyroxine; FT4, free tetraiodothyronine; TSH, thyroid-stimulating hormone. Bold values indicate statistically significant results.

**Table 3:** Correlations of 25(OH)D with various parameters.

Variable	*r*	*p* value	Variable	*r*	*p* value
Age (years)	0.030	0.329	FBG (mmol/L)	−0.031	0.314
Duration of DM (years)	0.032	0.294	OC (ng/ml)	−0.044	0.147
BMI (kg/m^2^)	−0.068	**0.026**	*β*-CTX (ng/ml)	−0.034	0.260
SBP (mmHg)	−0.060	**0.049**	P1NP (ng/ml)	−0.092	**0.003**
DBP (mmHg)	−0.008	0.782	PTH (pg/ml)	−0.179	**0.000**
Albumin (g/L)	0.247	**0.000**	Ca (mmol/L)	0.108	**0.000**
TC (mmol/L)	0.001	0.976	P (mmol/L)	−0.051	0.093
TG (mmol/L)	−0.069	**0.024**	TT3 (nmom/L)	0.124	**0.000**
LDL-C (mmol/L)	0.021	0.486	TT4 (nmom/L)	0.110	**0.000**
HDL-C (mmol/L)	0.067	**0.027**	FT3 (pmom/L)	0.143	**0.000**
ApoA1 (g/L)	0.079	**0.009**	FT4 (pmom/L)	0.075	**0.014**
Apob (g/L)	0.017	0.587	TSH (*μ*IU/mL)	0.009	0.766

BMI, body mass index; SBP, systolic blood pressure; DBP, diastolic blood pressure; TC, total cholesterol; TG, triglyceride; LDL-C, low-density lipoprotein cholesterol; HDL-C, high-density lipoprotein cholesterol; ApoA1, apolipoproteinA1; apob, apolipoprotein b; FBG: fasting blood glucose; OC, osteocalcin; *β*-CTX, *β*-C-terminal cross-linked telopeptide of type I collagen; P1NP, procollagen type 1 N-terminal propeptide; PTH, parathyroid hormone; Ca, calcium; P, phosphorus; TT3, triiodothyronine; TT4, tetraiodothyronine; FT3, free thyroxine; FT4, free tetraiodothyronine; TSH, thyroid-stimulating hormone. Bold values indicate statistically significant results.

**Table 4:** Adjusted association between HbA1c and 25(OH)D.

	HbA1c Level				
	B	95% CI	*β*	*t*	*p*-value
25(OH)D					
Model 1	−0.031	(−0.047, −0.014)	−0.108	−3.563	**0.000**
Model 2	−0.027	(−0.044, −0.010)	−0.094	−3.106	**0.002**
Model 3	−0.026	(−0.043, −0.008)	−0.091	−2.887	**0.004**

*Notes*. Data are expressed as partial regression coefficient (95% confidence interval). Model 1: crude model; model 2: adjusted for age, sex, BMI, and diabetes duration; model 3: additionally adjusted for albumin, SBP, TG, HDL-C, ApoA1, Ca, P, P1NP, PTH, TT3, TT4, FT3, and FT4. BMI, body mass index; SBP, systolic blood pressure; TG, triglyceride; HDL-C, high-density lipoprotein cholesterol; ApoA1, apolipoproteinA1; P1NP, procollagen type 1 N-terminal propeptide; PTH, parathyroid hormone; Ca, calcium; P, phosphorus; TT3, triiodothyronine; TT4, tetraiodothyronine; FT3, free thyroxine; FT4, free tetraiodothyronine. Bold values indicate statistically significant results.

## Data Availability

The data supporting the current study are available from the corresponding author (renluping1122@163.com).
